# Immediate Effects of Transcutaneous Spinal Stimulation on Stretch-Induced Spasticity in Persons with Spinal Cord Injury

**DOI:** 10.3390/brainsci15111201

**Published:** 2025-11-07

**Authors:** Evan B. Sandler, Jennifer A. Iddings, Edelle C. Field-Fote

**Affiliations:** 1 Crawford Research Institute, Shepherd Center, Atlanta, GA 30309, USA; evan.sandler@shepherd.org (E.B.S.); jennifer.iddings@shepherd.org (J.A.I.); 2 Division of Physical Therapy, School of Medicine, Emory University, Atlanta, GA 30322, USA; 3 Program in Applied Physiology, Georgia Institute of Technology, Atlanta, GA 30332, USA

**Keywords:** spinal cord injury, transcutaneous spinal stimulation, hyperreflexia, pendulum test, ankle clonus, spasticity, neuromodulation, biomechanics

## Abstract

**Background/Objectives**: Transcutaneous spinal stimulation (TSS) is a noninvasive stimulation approach for spasticity reduction in people with spinal cord injury (SCI). We enrolled 17 individuals with SCI who experience lower extremity hyperreflexia for this randomized crossover study to compare single-session effects of 3 TSS conditions: single-site continuous (SS-CONT), single-site burst (SS-BURST), and dual-site continuous (DS-CONT). **Methods**: Each TSS condition was delivered for 30 min with participants in supine via a cathode over the thoracic spine (T11–T12) and an anode over the abdomen. A second cathode was placed over the lumbar spine (L1/2 or L2/3) for DS-CONT. SS-CONT and DS-CONT stimulation was delivered as continuous 50 Hz stimulation with a 1 ms pulse width. SS-BURST stimulation was delivered as 4 bursts/second of 50 Hz stimulation with a 1 ms pulse width. Pendulum test first swing excursion (FSE) and ankle clonus drop test first drop excursion (FDE) were measured at baseline and immediately post-intervention to assess quadriceps and soleus spasticity, respectively. FSE and FDE of the first trial (FSE_T1_ and FDE_T1_) and the average of 3 trials (FSE_avg_ and FDE_avg_) were included in analyses. Subgroup analyses were performed based on baseline level of spasticity (high vs. low). **Results**: Between-condition analyses showed no significant differences; however, SS-CONT (FSE_T1_ *d* = 0.30, FSE_avg_ *d* = 0.27) and DS-CONT (FSE_T1_ *d* = 0.33, FSE_avg_ *d* = 0.12) stimulation demonstrated the largest effect sizes for FSE measures, and SS-CONT (FDE_T1_ *d* = 0.32, FDE_avg_ *d* = 0.31) stimulation demonstrated the largest effect size for FDE measures. Significant fair correlations between baseline FSE measures and change in FSE were identified when all conditions were combined. A significant fair correlation between baseline FDE_T1_ and change in FDE_T1_ was identified when data were collapsed across conditions. In subgroup analyses, only participants with high baseline quadriceps spasticity showed a significant decrease in quadriceps spasticity with DS-CONT (∆FSE_T1_ = 14.8 ± 13.0°), SS-BURST (∆FSE_T1_ = 4.1 ± 4.5°), and with all conditions combined (∆FSE_T1_ = 11.3 ± 16.5°, ∆FSE_avg_ = 7.2 ± 13.1°). For participants with low baseline soleus spasticity, DS-CONT stimulation significantly increased soleus spasticity (∆FDE_T1_ = −12.2 ± 9.3°, ∆FDE_avg_ = −8.5 ± 8.4°). **Conclusions**: When data were collapsed across conditions, TSS did not result in a significant reduction in quadriceps or soleus spasticity. Continuous stimulation at both single- and dual-sites was associated with the largest effect on quadriceps spasticity when all participants were combined. Lastly, TSS reduced spasticity in a severity-dependent manner.

## 1. Introduction

Spasticity is one of the hallmark features of spinal circuit dysregulation after spinal cord injury (SCI) [1]. Maladaptive changes in spinal cord circuitry result in excessive excitation to motoneurons, partly due to diminished influence from inhibitory circuits [2]. When activated, sensory afferents have a robust inhibitory influence on spinal circuits. After SCI, paresis and paralysis result in decreased movement and an associated reduction in movement-related afferent input.

Noninvasive electrical stimulation over the vertebrae, termed transcutaneous spinal stimulation (TSS), activates dorsal (posterior) spinal nerve roots [3]. TSS appears to have robust effects on spasticity and has the advantage that stimulation parameters can be customized or “tuned” to the individual [4,5,6]. However, optimal pulse parameters for spasticity reduction are not known. Despite the changes to spinal networks after SCI, spinal circuits continue to respond to temporal variations in sensory input. The majority of interventional TSS studies for spasticity reduction use a uniform 50 Hz frequency delivered continuously [7]. A recent in vivo computational modeling and preclinical study concluded that both 50 Hz continuous and patterned spinal stimulation promoted inhibition in hyperexcitable dorsal horn neurons, which likely contribute to pain and spasticity after SCI [8]. Moreover, neuromodulation studies at all levels of the neuraxis suggest benefits associated with the use of burst stimulation. In a comparison study, patterned afferent stimulation augmented inhibition within spinal reflex circuits, whereas uniform stimulation demonstrated no effect [9].

Beyond the temporal pattern of stimulation, another customizable parameter of TSS is the electrode location used to target lower extremity muscle activation. To date, studies of TSS have specified a single spinal level for cathodal stimulation, the T11/12 spinous interspace, with anodes placed anteriorly to direct current towards the spinal nerve roots [10]. The T11/12 electrode location preferentially activates the dorsal roots of the lumbar enlargement of the spinal cord, specifically, the afferents associated with monosynaptic activation of the motoneuron pools of the quadriceps muscles [11,12]. While spasticity of the quadriceps muscles is often problematic for persons with SCI, spasticity in the form of clonus of the soleus muscle can also be problematic and has been the target of numerous studies [13]. The addition of a second cathodal electrode over the L1/2 spinous interspace has the potential to modulate the more caudal area of the lumbar spinal cord, where the motoneuron pools for the soleus muscle are located [14].

The purpose of this study was to assess how TSS pulse parameters and electrode placement affect spasticity in participants with SCI who exhibit objectively measurable lower extremity spasticity. We assessed the immediate, within-session effects of 50 Hz continuous TSS delivered through electrodes placed at single and dual sites and 50 Hz burst TSS at a single site. The primary outcome of interest was change in quadriceps muscle spasticity as measured using the pendulum test first swing excursion (FSE). Change in soleus muscle spasticity as measured by the ankle clonus drop test, first drop excursion (FDE) was used as a secondary outcome to provide insight into the effects of an additional caudal lumbar electrode. Finally, we examined the relationship between baseline spasticity and TSS responsiveness to determine whether biomechanical measures of quadriceps and soleus hyperreflexia can serve as biomarkers to guide clinical decision-making.

## 2. Materials and Methods

This study was conducted with ethical approval from the Shepherd Center Research Review Committee. All participants gave their written informed consent prior to study enrollment, in accordance with the Declaration of Helsinki. This study was registered with clinicaltrials.gov (23 January 2020; NCT04243044). Complete details of the study methods are described in a prior publication, including acquisition of neurophysiologic reflex responses and interventional stimulation protocol [15].

### 2.1. Participants

Participants were enrolled if they met the following inclusion criteria: at least 16 years of age with SCI ≥ 3 months duration, regardless of injury severity, and presence of at least mild spasticity in either lower extremity. Spasticity was indicated by a pendulum test FSE ≤ 77°, or ≥5 beats of clonus on the ankle clonus drop test. Individuals with any of the following were excluded from participation: neurological level of injury below T12, progressive or potentially progressive spinal lesions, history of cardiovascular irregularities, active cancer or history of cancer, current pregnancy, implanted stimulators of any type, difficulty following instructions, or orthopedic pathology that would limit the ability to interpret study outcome measures (i.e., knee or hip flexion contractures > 10°).

At enrollment, the pendulum test and ankle clonus drop test were performed to identify the presence of spasticity. The most spastic lower extremity was determined as the lower extremity with the smaller pendulum test FSE, or if the participant did not exhibit quadriceps spasticity, as the lower extremity with the smaller ankle clonus drop test FDE. The same lower extremity was tested for all sessions. All participants were required to meet the spasticity inclusion criteria at the beginning of each session.

### 2.2. Study Design

This was a randomized, crossover study of a single session of each of three TSS conditions: single-site continuous stimulation (SS-CONT), single-site burst stimulation (SS-BURST), and dual-site continuous stimulation (DS-CONT). Biomechanical measures of quadriceps and soleus spasticity were captured before and immediately after the application of TSS to determine the effects of each TSS condition. There was a minimum of 48 h between sessions to reduce the potential for carryover effects.

### 2.3. Intervention

For the SS-CONT and SS-BURST conditions, stimulation was delivered through a single 5 cm round self-adhesive electrode (cathode) placed over the T11/12 spinous interspace. For the DS-CONT condition, stimulation was delivered through two 5 cm round self-adhesive electrodes (cathodes)—one over the T11/12 spinous interspace and one over either the L1/2 spinous interspace or the L2/3 spinous interspace. Two interconnected rectangular electrodes (5 × 9 cm each) were placed paraumbilically as anodes. SS-CONT stimulation: continuous and uniform 50 Hz charge-balanced, biphasic stimulation; SS-BURST stimulation: 4 bursts per second of 50 Hz pulses (130 ms train, 120 ms inter-train interval, 7 pulses/train) charge-balanced, biphasic stimulation; DS-CONT stimulation: continuous and uniform 50 Hz charge-balanced, biphasic stimulation at two locations as described above. An illustrative representation of the electrode configuration for all three conditions has been previously published [15].

All stimulation conditions (Vectra Genisys, Chattanooga/DJO, Carlsbad, CA, USA) were applied with a pulse width of 1 ms per phase for 30 min. Stimulation was delivered while participants remained in a supine position, with skin being checked intermittently for signs of adverse reactions.

### 2.4. Posterior Root-Muscle Reflex and Stimulation Intensity

Posterior root-muscle (PRM) reflexes were obtained to guide the stimulation intensity for each TSS condition. Electromyographic activity (EMG) was recorded from the soleus muscle using surface electrodes (MA300, Motion Lab Systems, Baton Rouge, LA, USA) and digitized (Power 1401, Cambridge Electronic Design, Cambridge, UK) at a sampling rate of 2 kHz using data capture software (Signal, Cambridge Electronic Design, Cambridge, UK).

Monophasic, rectangular pulses of 1 ms pulse width were delivered at the T11/12 interspinous space using a constant-current stimulator (Digitimer DS7AH, Hertforshire, UK). Pairs of stimuli with a 50 ms interstimulus interval (Grass S88X, Natus Neurology, Middleton, WI, USA) enabled verification of the reflex nature of the response through the presence of post-activation depression of the second stimulus response [11]. Reflex threshold (RT) was defined as the stimulation intensity that evoked responses in the soleus ≥ 100 μV peak-to-peak amplitude in at least 3 of 5 responses. Responses to five stimuli were collected at RT. TSS intensity was set to 0.8 × T or the highest intensity tolerable by the participant if they could not receive stimulation at 0.8 × RT.

### 2.5. Biomechanical Measurement of Spasticity

Within-session effects of TSS on spasticity were evaluated using biomechanical measurements of reflex-generated responses at the knee and ankle. Testing was completed before and immediately after TSS application.

The pendulum test assessed stretch-induced quadriceps spasticity [5,6,16,17,18]. Participants were positioned semi-reclined with the lower leg pendant over the edge of the mat and the shoe removed. To record knee angle during testing, an electrogoniometer (SG150, Biometrics Ltd., Newport, UK) was affixed to the test leg with the arms aligned with the midline of the thigh and lower leg, and the axis aligned with the knee joint center. The non-test leg was supported with the knee extended. Grasping the heel of the test leg, the examiner extended the knee and held the leg in this position for 60 s to allow movement-related excitability to dissipate. The examiner then released the heel, allowing the test leg to swing with gravity. FSE, the angle at which the knee first reversed from flexion to extension, was acquired and analyzed using Spike software (Cambridge Electronic Design Limited, Cambridge, UK). A larger FSE angle indicates less spasticity.

The ankle clonus drop test assessed stretch-induced soleus spasticity [19]. Participants were seated at the edge of a mat with knees at 90° flexion and back supported in a fully upright position to allow participants to relax their trunk and upper extremities during testing. The mat height was adjusted so the posterior surface of the upper leg was not touching the mat to allow sufficient distance between the mat and the leg when dropped. An electrogoniometer (SG150 or SG110, Biometrics Ltd., Newport, UK) was used to record the ankle angle of the test leg. The arms of the goniometer were aligned with the midline of the lower leg and the fifth metatarsal, and the axis was placed in the center of the ankle joint. Grasping the leg below the knee joint, the leg was lifted 10 cm above the resting position. The forefoot was positioned to strike the edge of a 6” platform when released, causing a stretch of the soleus. FDE, the angle at which the ankle first reversed from dorsiflexion to plantarflexion, was acquired and analyzed using Spike software (Cambridge Electronic Design Limited, Cambridge, UK). A larger FDE angle indicates less spasticity.

Previous literature has identified differences in biomechanical spasticity measurement due to repeated measurement [16,20,21]. Therefore, to obtain a comprehensive representation of stretch-induced quadriceps and soleus spasticity, the FSE and FDE of both the first trial (FSE_T1_ and FDE_T1_) and the average FSE and FDE of 3 trials (FSE_avg_ and FDE_avg_) of each test session were used for analysis.

### 2.6. Data Analysis

Analyses were executed using SPSS version 28 (IBM, London, UK). Data are presented as the mean ± SD. Between- and within-condition analyses were completed. When no significant between-condition differences were observed, data were collapsed across all conditions. To determine the effect of spasticity severity on responsiveness to TSS, participants were then subgrouped based on the median baseline FSE or FDE values. The subgroups were high-spasticity: FSE_avg_ < 59.0° and FSE_T1_ < 56.5°, FDE_avg_ < 38.4° and FDE_T1_ < 38.6°; and low-spasticity: FSE_avg_ ≥ 59.0° and FSE_T1_ ≥ 56.5°, FDE_avg_ ≥ 38.4° and FDE_T1_ ≥ 38.6°. Spasticity is known to vary daily [18]; therefore, it was necessary to subgroup participants for each outcome measure within a condition, as some participants who demonstrated high spasticity at baseline for one condition may have demonstrated low spasticity for other conditions, and vice versa. In each spasticity subgroup, between- and within-condition analyses were first completed. When no significant between-condition differences were observed within a subgroup, data were collapsed across all conditions.

All analyses were performed for FSE_avg_, FDE_avg_, FSE_T1_, and FDE_T1_. One-way ANOVAs were performed to determine differences between baseline values of the first, second, and third sessions and identify the presence of order effects. Paired *t*-tests were used to test for within-condition differences between pre- and post-intervention values for each condition. One-way ANOVAs were performed to determine differences between baseline values of the three stimulation conditions and to determine between-condition differences in change from baseline. Independent *t*-tests were used to identify differences in change from baseline between high- and low-spasticity subgroups. One-way ANOVAs were used to determine differences in change from baseline to post-intervention between conditions for the high- and low-spasticity subgroups. Post hoc Tukey tests were performed to identify any significant differences between the three conditions.

Effect sizes for pre-post change in each outcome measure were calculated using Cohen’s *d* based on the pooled variance of the compared values. Effect sizes were categorized as small (0.2), moderate (0.5), or large (0.8) [22]. Effect sizes offer insight into the magnitude of the outcome regardless of statistical significance and allow comparison between intervention conditions regarding change [23]. Comparison between conditions allows meaningful decisions for research translation into clinical practice.

Pearson correlations (*r*) were calculated to determine the relationship between change scores and baseline measures for each TSS condition and when data were collapsed across conditions. Pearson correlations were also calculated to determine the relationship between measures of knee and ankle spasticity. Designations of correlation coefficients were no relationship (<0.25), fair (0.26–0.5), moderate (0.51–0.75), and good (>0.76) [24]. For all analyses, significance was set at α = 0.05. Increases in FSE and FDE are represented as positive values, indicating increased joint angles (i.e., decrease in spasticity), whereas decreases are represented by negative values, indicating decreased joint angles (i.e., increase in spasticity). All data are presented first as FSE_avg_ or FDE_avg_, followed by FSE_T1_ or FDE_T1_.

## 3. Results

Out of 21 participants enrolled, usable data were obtained from 17 participants. Individual movement limitations restricted the collection of all outcome measures for the 17 participants. Of the 17 participants with usable data, 13 participants completed all three conditions, 2 participants completed 2 conditions, and 2 participants completed only one condition. Participant randomization and withdrawal can be found in the previously published CONSORT diagram [15]. Participant demographics have been previously published [15].

Of the 82 instances in which the pendulum test was performed, accounting for all participants, conditions, and timepoints, we observed the smallest FSE (i.e., highest quadriceps spasticity) in 46 instances for trial one, 19 for trial two, and 17 for trial three. Of the 90 instances in which the ankle clonus drop test was performed, we observed the smallest FDE (i.e., highest soleus spasticity) in 22 instances for trial one, 33 for trial two, and 35 for trial three.

No order effects for session were identified for FSE_avg_, FSE_T1_, FDE_avg_, or FDE_T1_, respectively (F(2,38) = 0.52 *p* = 0.60, F(2,38) = 0.68 *p* = 0.51, F(2,42) = 1.74 *p* = 0.19, F(2,42) = 1.76 *p* = 0.18).

### 3.1. Stretch-Induced Spasticity of the Quadriceps

#### 3.1.1. Whole Group Analysis

**Figure 1:**
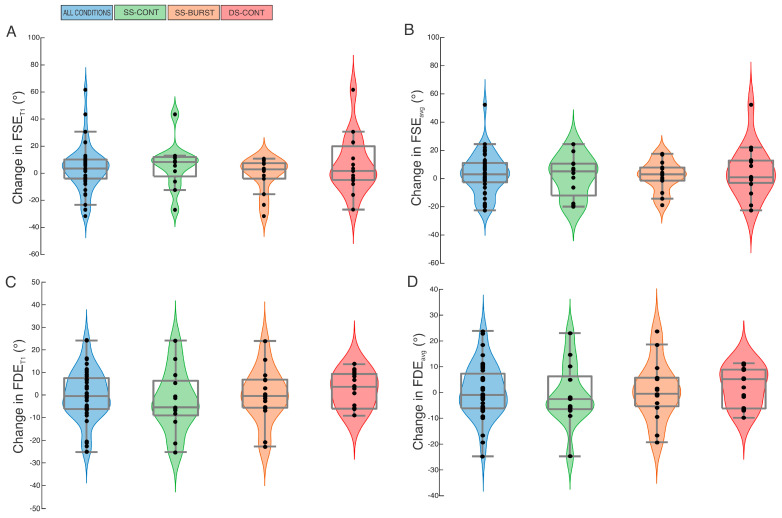
Violin plots reporting change in FSE_T1_ (**A**), FSE_avg_ (**B**), FDE_T1_ (**C**), and FDE_avg_ (**D**) for all conditions combined (blue), SS-CONT (green), SS-BURST (orange), and DS-CONT (red). Small blue dots represent individual data points.

At baseline, no differences were found for FSE_avg_ or FSE_T1_ among SS-CONT, DS-CONT, and SS-BURST (F(2,38) = 0.16, *p* = 0.86, F(2,38) = 0.02, *p* = 0.98) (Table 1). Between-condition analysis identified no difference in pre-post change in FSE_avg_ or FSE_T1_ between the three intervention conditions (F(2,38) = 0.21, *p* = 0.81; F(2,38) = 0.86, *p* = 0.43). Within-condition pre-post analysis showed all conditions demonstrated small or no effect and non-significant increases in FSE_avg_ and FSE_T1_ after intervention, except SS-BURST for the FSE_T1_ comparison (Table 2, Figure 1A,B). SS-BURST demonstrated a non-significant decrease in FSE_T1_ with no effect. The largest effect size for change in FSE_avg_ was demonstrated after SS-CONT stimulation; the largest effect size for change in FSE_T1_ was observed after DS-CONT stimulation (Table 2, Figure 1A,B). When data were collapsed across intervention conditions, there was no significant change in FSE_avg_ or FSE_T1_ (Table 2, Figure 1A,B).

#### 3.1.2. High-Spasticity Subgroup Analysis

The mean baseline FSE_avg_ and FSE_T1_ for the high-spasticity subgroup were 45.8 ± 7.3° and 43.8 ± 7.0°, respectively. Between-condition analysis showed no difference in the change in mean FSE_avg_ and FSE_T1_ between intervention conditions (FSE_avg_: F(2,18) = 1.0, *p* = 0.38; FSE_T1_: F(2,18) = 1.03, *p* = 0.38). Within-condition analysis showed large, significant increases in mean FSE_T1_ after DS-CONT and SS-BURST stimulation (Table 3). Although non-significant, there was a moderate effect after SS-CONT stimulation for FSE_avg_ and FSE_T1_ (Table 3). When data were collapsed across intervention conditions, moderate and significant increases were observed in both mean FSE_avg_ and FSE_T1_ (Table 3).

#### 3.1.3. Low-Spasticity Subgroup Analysis

The mean baseline FSE_avg_ and FSE_T1_ for the low-spasticity subgroup were 81.2 ± 17.6° and 79.9 ± 20.3°, respectively. Between-conditions analysis showed no difference in the change in mean FSE_avg_ and FSE_T1_ between intervention conditions (FSE_avg_: F(2,17) = 0.43, *p* = 0.66; FSE_T1_: F(2,17) = 0.33, *p* = 0.72). Within-condition analysis identified DS-CONT stimulation demonstrated the largest mean decrease in FSE_avg_ and FSE_T1_ with small and moderate effects, respectively (Table 3). When data were collapsed across intervention conditions, mean FSE_avg_ and FSE_T1_ demonstrated no significant decrease (Table 3).

### 3.2. Stretch-Induced Spasticity of the Soleus

#### 3.2.1. Whole Group Analysis

At baseline, no differences were found for FDE_avg_ or FDE_T1_ among the three intervention conditions (F(2,42) = 1.98, *p* = 0.15; F(2,42) = 2.01, *p* = 0.15) (Table 1). Between-condition analysis identified no difference in pre-post change in FDE_avg_ and FDE_T1_ between the three intervention conditions (F(2,42) = 0.37, *p* = 0.69; F(2,42) = 0.70, *p* = 0.50). Within-condition pre-post analysis showed no significant change with small or no effect in FDE_avg_ or FDE_T1_ for any condition (Table 2, Figure 1C,D). SS-CONT demonstrated the largest effect size, albeit small, for both change in FDE_avg_ and FDE_T1_ (Table 2, Figure 1C,D). When data were collapsed across intervention conditions, there was no significant change in FDE_avg_ or FDE_T1_ (Table 2, Figure 1C,D).

#### 3.2.2. High-Spasticity Subgroup Analysis

The mean baseline FDE_avg_ and FDE_T1_ for the high-spasticity subgroup were 33.1 ± 4.5° and 33.6 ± 4.6°, respectively. There were no significant differences in change between conditions for FDE_avg_ or FDE_T1_ (F(2,20) = 0.49, *p* = 0.62; F(2,20) = 0.60, *p* = 0.56). Within-condition analysis showed the largest mean increases in FDE_avg_ and FDE_T1_ with moderate effect were observed after DS-CONT stimulation (Table 3). Mean FDE_avg_ and FDE_T1_ also demonstrated a small non-significant increase after SS-CONT stimulation (Table 3). No significance or effect was demonstrated after SS-BURST stimulation (Table 3). When data were collapsed across intervention conditions, a small, non-significant increase in both mean FDE_avg_ and FDE_T1_ was observed (Table 3).

#### 3.2.3. Low-Spasticity Subgroup Analysis

The mean baseline FDE_avg_ and FDE_T1_ for the low-spasticity subgroup were 45.7 ± 7.0° and 46.7 ± 8.1°, respectively. Between-condition analysis showed significant differences in change for FDE_T1_ only (FDE_avg_: F(2,19) = 2.53, *p* = 0.10; FDE_T1_ F(2,19) = 4.00, *p* = 0.04). Post hoc comparisons revealed significant differences between SS-CONT and DS-CONT stimulation only (FDE_T1_: *p* = 0.03). Within-condition analysis shows SS-CONT stimulation demonstrated a small, non-significant increase in FDE_avg_ and FDE_T1_ (Table 3). DS-CONT stimulation demonstrated a large, significant decrease in both FDE_avg_ and FDE_T1_ (Table 3). When data were collapsed across intervention conditions, no change was observed in mean FDE_avg_ and FDE_T1_ (Table 3).

### 3.3. Correlations of Baseline Spasticity and Change in Biomechanical Measures

**Figure 2:**
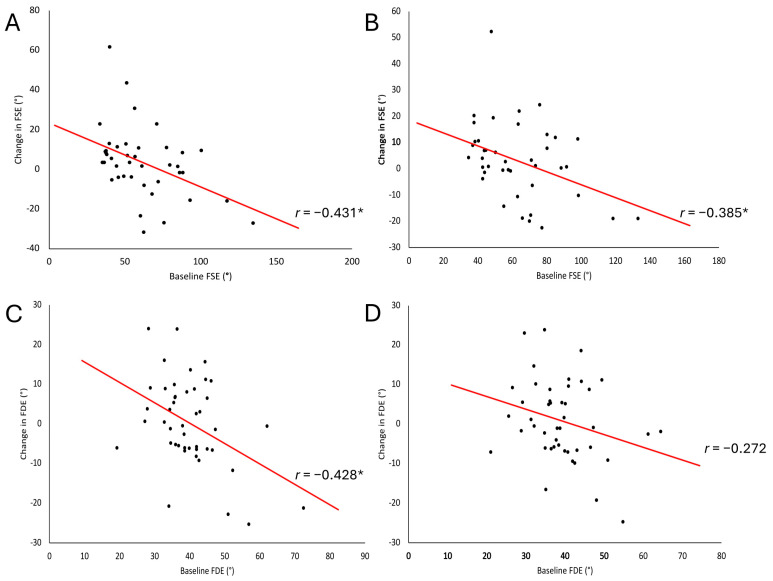
Change in quadriceps and soleus spasticity as a function of individuals’ baseline spasticity. Correlations of baseline FSE_T1_ (**A**), FSE_avg_ (**B**), FDE_T1_ (**C**), and FDE_avg_ (**D**) to change in the respective measure are presented for the whole group. Significance (*p* < 0.05) of the Pearson correlation coefficient is indicated with an asterisk (*).

Participants with higher baseline spasticity (smaller FSE and FDE) experienced greater change, as indicated by negative correlations when data were collapsed across intervention conditions. Correlations between baseline FSE_avg_ and FSE_T1_ and change in the respective measures were fair and significant when data were collapsed across intervention conditions (Figure 2A,B). Within-condition analysis demonstrated a fair and significant relationship in the SS-CONT condition between baseline FSE_T1_ and change in FSE_T1_ and a moderate and significant relationship for the DS-CONT condition (Table 4). In addition, baseline FSE_avg_ and change in FSE_avg_ demonstrated a significant relationship in the DS-CONT condition only, which was moderate (Table 4). Baseline FDE_avg_ and change in FDE_avg_ demonstrated no relationship (Figure 2D) when data were collapsed across intervention conditions. Within-condition analysis demonstrated a moderate and significant relationship between baseline FDE_avg_ and change in FDE_avg_ (Table 4). Correlation between baseline FDE_T1_ and change in FDE_T1_ was fair and significant (Figure 2C) when data were collapsed across intervention conditions and moderate and significant in the DS-CONT condition. No relationships were found between measures of spasticity at the knee and the ankle at baseline. No relationships were found between measures of change in spasticity post-intervention at the knee and the ankle.

## 4. Discussion

This study compared the single-session effects of three TSS conditions (SS-CONT, DS-CONT, and SS-BURST) to assess their influence on spasticity in participants with SCI who experience hyperreflexia of the muscles about the knee and/or ankle. Biomechanical measures of stretch-induced reflex excitability were used to assess spasticity. Quadriceps spasticity did not change significantly from baseline to post-TSS intervention in any of the conditions when data from the whole group were analyzed together. The largest effect sizes for change in quadriceps spasticity post-intervention were for the SS-CONT condition for FSE_avg_ and for the DS-CONT condition for FSE_T1_. In whole group analysis, the largest decrease in soleus spasticity from baseline to post-TSS intervention was observed in the SS-CONT condition. Moreover, the change in soleus spasticity after SS-CONT was associated with small effect sizes for both measures, FDE_avg_ and FDE_T1_.

### 4.1. Effects of Baseline Spasticity on Responsiveness to TSS

Prior studies have shown that responsiveness to afferent stimulation is influenced by the baseline level of spasticity [6,18]. The results of the current study are consistent with those findings. When combining all participants and conditions, there was a significant fair relationship between baseline quadriceps spasticity (FSE_avg_ and FSE_T1_) and the magnitude of change post-intervention. For soleus spasticity, there was also a significant fair relationship between baseline FDE_T1_ and change post-intervention; however, there was no relationship between baseline FDE_avg_ and magnitude of change post-intervention when data were collapsed across conditions. In addition, with all intervention conditions combined, the high-spasticity subgroup (FSE_avg_ < 59.0° and FSE_T1_ < 56.5°) demonstrated a significant decrease in quadriceps spasticity, whereas there was no change in the low-spasticity subgroup (FSE_avg_ ≥ 59.0° and FSE_T1_ ≥ 56.5°). In the high-spasticity subgroup, spasticity decreased significantly for DS-CONT and SS-BURST. There was no significant change in soleus spasticity in the high-spasticity subgroup (FDE_avg_ < 38.4° and FDE_T1_ < 38.6°) or the low-spasticity subgroup (FDE_avg_ ≥ 38.4° and FDE_T1_ ≥ 38.6°) from baseline to post-intervention. DS-CONT stimulation significantly increased soleus spasticity in the low-spasticity subgroup.

### 4.2. Differences in Current Delivery Between Conditions

Differences in total current delivered during spinal stimulation may account for differences observed between intervention conditions. Stimulation intensity for each condition was maintained below motor threshold to activate only large-diameter afferent fibers. Electrical fields targeting spinal circuits have been shown to modulate firing patterns of spinal neurons in an intensity dose-dependent manner [25,26]. Although intensity was consistently 0.8 × RT for each condition, DS-CONT stimulation was delivered at 0.8 × RT at each of the two electrodes, effectively doubling the total charge delivered. Moreover, SS-BURST stimulation included “off” time, thereby reducing the total charge delivery in comparison to continuous stimulation.

The SS-BURST condition demonstrated less effect on quadriceps spasticity than SS-CONT for within-condition, whole-group analyses. This is not consistent with previous literature demonstrating a greater inhibitory effect on mechanisms of spasticity after patterned stimulation compared to uniform/continuous stimulation [9]. However, SS-BURST stimulation demonstrated a large, significant positive effect for FSE_T1_ for the high spasticity subgroup in contrast to a small, non-significant negative effect in the low spasticity subgroup. Stimulation pattern, therefore, had an influence on neural circuits that was not evident until participants were stratified into high and low spasticity subgroups. Of note, the total charge in the SS-BURST condition was less than that of the SS-CONT and DS-CONT conditions. Taken together, this may indicate that the introduction of a pattern into the stimulation may preferentially activate inhibitory spinal circuits responsible for quadriceps spasticity reduction, but only in individuals with high spasticity, and may require less overall charge delivery to demonstrate effect.

### 4.3. Dual-Site Electrodes Preferentially Target the Soleus

Preferential activation of spinal circuits can be achieved, dependent upon the level of the spinal cord at which stimulation is applied [14]. The probability of activating circuits associated with the quadriceps is greatest at L2–L4 spinal levels and S1/2 for the soleus [14]. Whole group analyses demonstrated a decrease in ankle spasticity after SS-CONT stimulation. This suggests the SS-CONT condition was capable of modulating more caudal dorsal nerve roots, most likely through current dispersion at multiple spinal levels [27]. The beneficial effects of SS-CONT stimulation on ankle spasticity, as indicated by a small effect size (FSE_avg_ *d* = 0.23, FSE_T1_ *d* = 0.38), contrast with results from previous work, potentially due to methodological differences [28]. Estes et al. combined locomotor training and continuous spinal stimulation and found no effect, as indicated by an effect size of *d* = 0.06 on soleus spasticity of the more impaired limb as measured by the number of oscillations in the ankle clonus drop test.

In the high spasticity subgroup, DS-CONT stimulation resulted in decreased ankle spasticity with a moderate effect size. The small increase in soleus spasticity observed in the whole group analysis of the DS-CONT condition may be attributable to the high variability in baseline spasticity when all participants were combined. Subsequently, subgroup analysis revealed participants with high spasticity at the soleus benefited from the addition of the second electrode, as demonstrated by a larger effect on FDE_avg_ and FDE_T1_ after DS-CONT stimulation compared to SS-CONT stimulation. In contrast, there was a significant increase in spasticity in participants with low spasticity after DS-CONT stimulation. Afferent fibers associated with the soleus were likely recruited with the addition of a lumbar electrode for dual-site stimulation, promoting neuromodulation of soleus spinal circuits.

### 4.4. Activation of Multisegmental Inhibitory Circuits

Neuronal connections between the rostral and caudal motoneuron pools are responsible for excitation and inhibition of circuits that regulate interlimb and intralimb timing [29,30,31,32,33]. Specifically, the spinal networks related to the quadriceps exert heteronymous influence on the soleus in humans [33,34]. An inhibitory effect of rostral spinal circuits on caudal spinal circuits, dependent upon temporal characteristics of each stimulus, has also been demonstrated during dual-site stimulation [34]. Moreover, single-site stimulation at either thoracic or lumbar electrode locations resulted in larger evoked responses and corresponding muscle forces at proximal and distal muscles compared to multi-site stimulation [35]. In our study, each electrode (thoracic and lumbar) provided 50 Hz stimulation independently. The relative timing (rostral followed by caudal or caudal followed by rostral) of stimulating pulses from each of the two electrodes in the DS-CONT was therefore not consistent, potentially producing varying timing of activation between rostral (quadriceps) and caudal (soleus) spinal circuits. Single-site stimulation (SS-CONT) resulted in the largest decrease in ankle spasticity with all participants combined. Conversely, dual-site stimulation (DS-CONT) resulted in no significant change with all participants combined or in the high-spasticity subgroup and a significant increase in ankle spasticity in the low-spasticity subgroup. It is possible that spatiotemporal-dependent characteristics of dual-site stimulation, i.e., location and timing of stimulation at each electrode, may account for the lack of significant effect observed in the DS-CONT condition.

### 4.5. Delayed Effect of TSS on Quadriceps and Soleus Spasticity

The magnitude of TSS effects depends on the timing of post-intervention measurement [6,18]. A previous study of the persistent effects of a single session of TSS demonstrates a significant decrease in quadriceps spasticity for participants categorized as having high spasticity immediately following and 45 min post-intervention [6]. The stimulation in these studies was delivered continuously at a single site at a frequency of 50 Hz with a pulse width of 400 μs for 15 min. However, the antispasmodic effect was non-significant at the 15-min post-intervention measurement time point. The biomechanical measurements of spasticity, the pendulum test and the ankle clonus drop test, were assessed in the current study at a single time point, approximately 15–30 min post-intervention. Due to the post-assessment timing in the current study, the previously observed time-dependent effect may account for the lack of significance in the change in spasticity of the quadriceps found in post-intervention measurement for the single-site (SS-CONT and SS-BURST) conditions. It is likely that the timing of the post-TSS assessment would have a similar influence on outcomes at the quadriceps and at the soleus.

### 4.6. TSS Overcomes Immobility-Induced Spasticity

Therapeutic interventions that involve activation of sensory afferents through movement, as well as devices that stimulate these same afferents, have been shown to reduce spasticity [5]. By comparison, imposed immobility of participants remaining in a supine position for the 30-min duration of sham stimulation resulted in increased spasticity [5]. Therefore, the anti-spasmodic effect of spinal stimulation must overcome the spasticity-inducing effects of immobility. While no sham condition was included in the current study, the low-spasticity subgroup may have experienced the negative effects of immobility during intervention. Movement and activity are known to have antispasmodic effects [5,36]. Multiple studies of spinal stimulation for motor function have combined the intervention with task practice [28,37,38]. For this reason, it may be advisable to combine spinal stimulation with movement-based interventions to augment its antispasmodic effects.

### 4.7. Limitations

This study serves as a foundation to identify appropriate dosing parameters for the use of spinal stimulation to reduce spasticity. However, several limitations must be addressed to assist in the clinical translation of spinal stimulation used in this study. First, individuals with spasticity at the knee or the ankle were included in this study. Participants were not required to demonstrate spasticity at both joints. At enrollment and prior to each session, individuals who exhibited spasticity at the knee may not have exhibited spasticity at the ankle, and vice versa. Despite this, measures were taken at both joints during each session, which may explain the lack of relationship found between spasticity of the knee and ankle in this study. Second, the pendulum test and ankle clonus drop test measure a single component of spasticity, hyperreflexia, around a single joint in isolation. In a survey of people with tetraplegia and paraplegia after SCI, stiffness was reported to be more problematic than spontaneous spasms and hyperreflexia for both levels of paresis [39]. Clinical measurement of spasticity presentation is limited to assessment of stretch-induced ordinal measures; further research is needed to quantify qualities of spasticity that have the greatest impact on lived experience. The current study did not assess the persistence of any anti-spasmodic effect beyond the single immediate post-intervention time point. Identifying therapeutic interventions to reduce dependence upon pharmaceuticals, which are often associated with negative side effects, requires an understanding of the duration of effects. Moreover, this was a single-session study that used a stimulation intensity below motor threshold. Further research is needed to elaborate upon the potential benefits of multi-session use of spinal stimulation applied at various intensities to establish its efficacy with consistent use in a clinical or home setting.

## 5. Conclusions

In the current study, we identified that when data were collapsed across conditions, TSS did not result in a significant reduction in quadriceps or soleus spasticity. Continuous stimulation at both single- and dual-sites, SS-CONT and DS-CONT, respectively, were associated with the largest effect on quadriceps spasticity when all participants were combined. Based on our findings, the metric used to assess quadriceps and soleus spasticity influences the study outcome. We have demonstrated that the TSS condition with the largest effect on quadriceps spasticity differs when using the first trial or the average of three trials of the pendulum test. We recommend the use of the average of three trials when assessing quadriceps spasticity via the pendulum test, as the first trial did not always result in the smallest FSE. Although the TSS condition with the largest effect on soleus spasticity did not differ when using the first trial or the average of three trials of the ankle clonus test, we recommend the use of the average of three trials when assessing soleus spasticity via the ankle clonus drop test. By using the average of three trials, the known variability of spasticity can be accounted for.

TSS was demonstrated to reduce spasticity in a severity-dependent manner. Identification of spasticity severity at the knee and ankle is therefore critical prior to TSS application. We recommend the use of SS-CONT stimulation when unable to obtain an objective measurement of spasticity severity. Although DS-CONT and SS-CONT stimulation demonstrated the largest effect on quadriceps spasticity when low and high spasticity subgroups were considered together, DS-CONT stimulation, however, resulted in an increase in spasticity in the low spasticity subgroup. If spasticity severity is known to be high, we recommend the use of DS-CONT stimulation for reduction in quadriceps spasticity. We also recommend the use of DS-CONT stimulation when targeting spasticity of the soleus, but only when an individual presents with high spasticity severity.

## Figures and Tables

**Table 1:** Baseline values for all conditions combined and each condition.

	ALL CONDITIONS	SS-CONT	SS-BURST	DS-CONT
FSE_T1_ (°)	61.4 ± 23.5	61.2 ± 22.1	60.7 ± 21.5	62.5 ± 29.0
FSE_avg_ (°)	63.4 ± 22.9	65.0 ± 22.5	60.6 ± 20.6	64.8 ± 27.4
FDE_T1_ (°)	40.0 ± 9.2	37.2 ± 7.7	39.6 ± 5.1	43.9 ± 13.3
FDE_avg_ (°)	39.2 ± 8.6	36.8 ± 8.0	38.7 ± 5.0	42.9 ± 11.6

**Table 2:** Change in quadriceps and soleus spasticity for all conditions combined and each condition. FSE = first swing excursion, FDE = first drop excursion. Cohen’s *d* effect size of change for each measure is indicated in parentheses.

	ALL CONDITIONS	SS-CONT	SS-BURST	DS-CONT
∆FSE_T1_ (°)	3.5 ± 17.6 (0.20) *n* = 41	6.4 ± 21.5 (0.30) *n* = 15	−1.5 ± 13.0 (−0.12) *n* = 14	5.7 ± 16.9 (0.33) *n* = 12
∆FSE_avg_ (°)	3.0 ± 14.7 (0.20) *n* = 41	5.0 ± 18.3 (0.27) *n* = 15	1.9 ± 10.7 (0.18) *n* = 14	1.7 ± 14.7 (0.12) *n* = 12
∆FDE_T1_ (°)	0.2 ± 11.1 (0.02) *n* = 45	2.4 ± 7.6 (0.32) *n* = 17	0.0 ± 12.1 (0.00) *n* = 15	−2.4 ± 13.8 (−0.18) *n* = 13
∆FDE_avg_ (°)	0.8 ± 10.2 (0.08) *n* = 45	2.3 ± 7.4 (0.31) *n* = 17	0.6 ± 11.5 (0.05) *n* = 15	−0.9 ± 12.0 (−0.08) *n* = 13

**Table 3:** Change in quadriceps and soleus spasticity by spasticity subgroup for all conditions combined and each condition. Significance (*p* < 0.05) in pre-post change using paired *t*-tests is indicated with an asterisk (*). Cohen’s *d* effect size of change for each measure is indicated in parentheses.

	ALL CONDITIONS		SS-CONT		SS-BURST		DS-CONT	
	HIGH	LOW	HIGH	LOW	HIGH	LOW	HIGH	LOW
∆FSE_T1_ (°)	11.3 ± 16.5 * (0.69) *n* = 21	−4.8 ± 15.0 (−0.32) *n* = 20	15.2 ± 25.0 (0.61) *n* = 7	−1.3 ± 15.7 (−0.08) *n* = 8	4.1 ± 4.5 * (0.91) *n* = 7	−7.1 ± 16.6 (−0.43) *n* = 7	14.8 ± 13.0 * (1.14) *n* = 7	−7.1 ± 13.6 (−0.52) *n* = 5
∆FSE_avg_ (°)	7.2 ± 13.1 * (0.55) *n* = 21	−1.5 ± 15.2 (−0.10) *n* = 20	11.1 ± 20.0 (0.56) *n* = 7	−0.4 ± 15.9 (−0.03) *n* = 8	2.0 ± 8.9 (0.23) *n* = 8	1.8 ± 13.6 (0.13) *n* = 6	9.7 ± 5.4 (0.78) *n* = 6	−6.3 ± 17.1 (−0.37) *n* = 6
∆FDE_T1_ (°)	2.6 ± 10.2 (0.26) *n* = 23	−2.3 ± 11.7 (−0.20) *n* = 22	2.3 ± 6.2 (0.38) *n* = 7	2.5 ± 8.7 (0.29) *n* = 10	0.2 ± 12.1 (0.02) *n* = 9	−0.4 ± 13.3 (−0.03) *n* = 6	6.0 ± 11.3 (0.53) *n* = 7	−2.2 ± 9.3 * (−1.13) *n* = 6
∆FDE_avg_ (°)	2.5 ± 9.6 (0.26) *n* = 23	−1.0 ± 10.6 (−0.10) *n* = 22	1.5 ± 6.7 (0.23) *n* = 7	2.8 ± 8.2 (0.35) *n* = 10	0.9 ± 10.9 (0.08) *n* = 9	0.1 ± 13.4 (0.00) *n* = 6	5.6 ± 11.0 (0.51) *n* = 6	−8.5 ± 8.4 * (−1.02) *n* = 7

**Table 4:** Within-condition correlations of baseline FSE and FDE measures and the change in the respective measure. Significant (*p* < 0.05) Pearson correlations (*r*) are indicated with an asterisk (*).

	SS-CONT	SS-BURST	DS-CONT
FSE_T1_ (°)	−0.45 *	−0.20	−0.65 *
FSE_avg_ (°)	−0.43	−0.13	−0.55 *
FDE_T1_ (°)	0.14	−0.19	−0.75 *
FDE_avg_ (°)	0.24	−0.19	−0.59 *

## Data Availability

The data supporting the conclusions of this article will be made available by the authors on request.

## Abbreviations

The following abbreviations are used in this manuscript:

TSS	Transcutaneous spinal stimulation
SCI	Spinal cord injury
PRM	Posterior root-muscle
SS-CONT	Single-site continuous
SS-BURST	Single-site burst
DS-CONT	Dual-site continuous
RT	Reflex threshold
FDE	First drop excursion
FSE	First swing excursion
RA	Response amplitude
EMG	Electromyography
